# Calcaneus Wound Healing With the Aid of Urinary Bladder Matrix (UBM)

**DOI:** 10.7759/cureus.117954

**Published:** 2026-10-15

**Authors:** Tareq Alalwan, Mohammed Tarabishi, Lotus Akkad, Ghadeer Alnojaidi, Yara A Alalwan

**Affiliations:** 1 Orthopedic Surgery, King Fahad Medical City, Riyadh, SAU; 2 Pharmacology, Jordan University of Science and Technology, Irbid, JOR; 3 Medicine, King Saud University, Riyadh, SAU

**Keywords:** antimicrobial peptide, calcaneus fracture, ecm, postoperative wound, type 2 macrophage, ubm

## Abstract

This case report examines the use of urinary bladder matrix (UBM) in the management of complex wounds, specifically an infected calcaneal wound that developed following open reduction and internal fixation (ORIF). The wound was classified as acute and contaminated, with devitalized tissue present. Wound-healing complications are recognized following calcaneal fracture surgery. The primary aim of this report was to achieve a favorable clinical outcome while minimizing treatment costs.

A 43-year-old man presented to the outpatient department with an infected, discolored wound over the right lateral calcaneus following ORIF. He was subsequently admitted to the hospital for urgent excisional debridement of devitalized wound-bed tissue and application of UBM-based xenograft regenerative materials (MicroMatrix® and Cytal®, Integra LifeSciences Corp., Princeton, NJ, USA).

UBM is an extracellular matrix (ECM)-based device that may promote tissue remodeling and modulate inflammation at the wound site. It may also exhibit antimicrobial activity through bioactive peptides generated by the proteolysis of collagen and elastin within the ECM.

## Introduction

Calcaneal fractures, or fractures of the heel bone, are the most common fractures of the tarsal bones, accounting for approximately 2% of all fractures and 60%-65% of tarsal fractures [1,2]. Their management is particularly challenging because of the complex anatomy of the calcaneus, its weight-bearing function, and the limited soft tissue coverage surrounding the bone [3]. Open reduction and internal fixation (ORIF) is a commonly used surgical treatment for displaced calcaneal fractures. According to Dhillon et al. [3], ORIF enables many patients to regain pain-free weight-bearing function, representing an important milestone in recovery [4].

Calcaneal fractures typically result from high-energy trauma, such as falls from significant heights or motor vehicle accidents. These injuries may cause widening and loss of height of the heel, disruption of the subtalar joint surface, and malalignment of the hindfoot [5,6]. Although these fractures are rarely life-threatening, they can result in substantial long-term disability if not managed appropriately [4].

Despite the potential for favorable functional outcomes following ORIF, the procedure carries a considerable risk of complications. A retrospective study involving 176 patients with closed intra-articular calcaneal fractures reported an overall postoperative complication rate of approximately 27%. Reported complications included wound infections in 7 patients (3.9%), comprising deep infections (1.7%) and superficial infections (2.3%); soft tissue necrosis in 12 patients (6.8%); postoperative pain in 14 patients (8.0%), including pain associated with arthritis, heel pad discomfort, and generalized heel pain; malunion in 5 patients (2.8%); nonunion in 2 patients (1.1%); loss of fixation in 4 patients (2.3%); and nerve injuries in 3 patients (1.7%). No cases of compartment syndrome were reported [4].

Despite these potential complications, ORIF remains an important treatment option for displaced calcaneal fractures, as it can restore anatomical alignment and may improve functional outcomes and pain relief in appropriately selected patients. However, the risk of postoperative complications highlights the importance of careful patient selection, meticulous surgical technique, and close postoperative monitoring.

## Case presentation

A 43-year-old man with no significant past medical history presented to the Emergency Department in February after falling approximately 4 m while working in the field and landing on his right foot. His medical history was notable for heavy tobacco use, amphetamine and cannabis abuse, and appendectomy 10 years earlier.

The patient reported severe right ankle pain and tenderness. He denied head trauma, loss of consciousness, nausea, vomiting, seizures, or headache.

On physical examination, the patient was alert, oriented, and hemodynamically stable, with vital signs within normal limits. Examination of the right ankle revealed marked swelling, tenderness, painful range of motion, and soft tissue injury. Initial neurovascular assessment demonstrated preserved distal perfusion and sensation.

Initial trauma investigations, including pelvic radiography and an extended Focused Assessment with Sonography for Trauma (eFAST), revealed no additional injuries. Plain radiographs of the right ankle demonstrated a displaced, comminuted calcaneal fracture with intra-articular extension. Computed tomography (CT), performed in March 2025, confirmed a displaced, comminuted intra-articular calcaneal fracture with multiple fragments.

The patient underwent open reduction and internal fixation (ORIF) on under general anesthesia with a regional nerve block. The articular surface was reduced to achieve satisfactory joint congruity. Fixation was performed using two Kirschner wires, two compression screws, and a lateral locking plate secured with cortical and locking screws. Postoperatively, distal perfusion remained intact, with normal capillary refill. The patient was discharged in April 2025, with instructions to remain non-weight-bearing and attend an outpatient follow-up appointment two weeks later.

At follow-up in May 2025, the patient presented with clinical signs of a surgical site infection (SSI), including wound discoloration, purulent discharge, and a foul odor (Figure 1).

**Figure 1:**
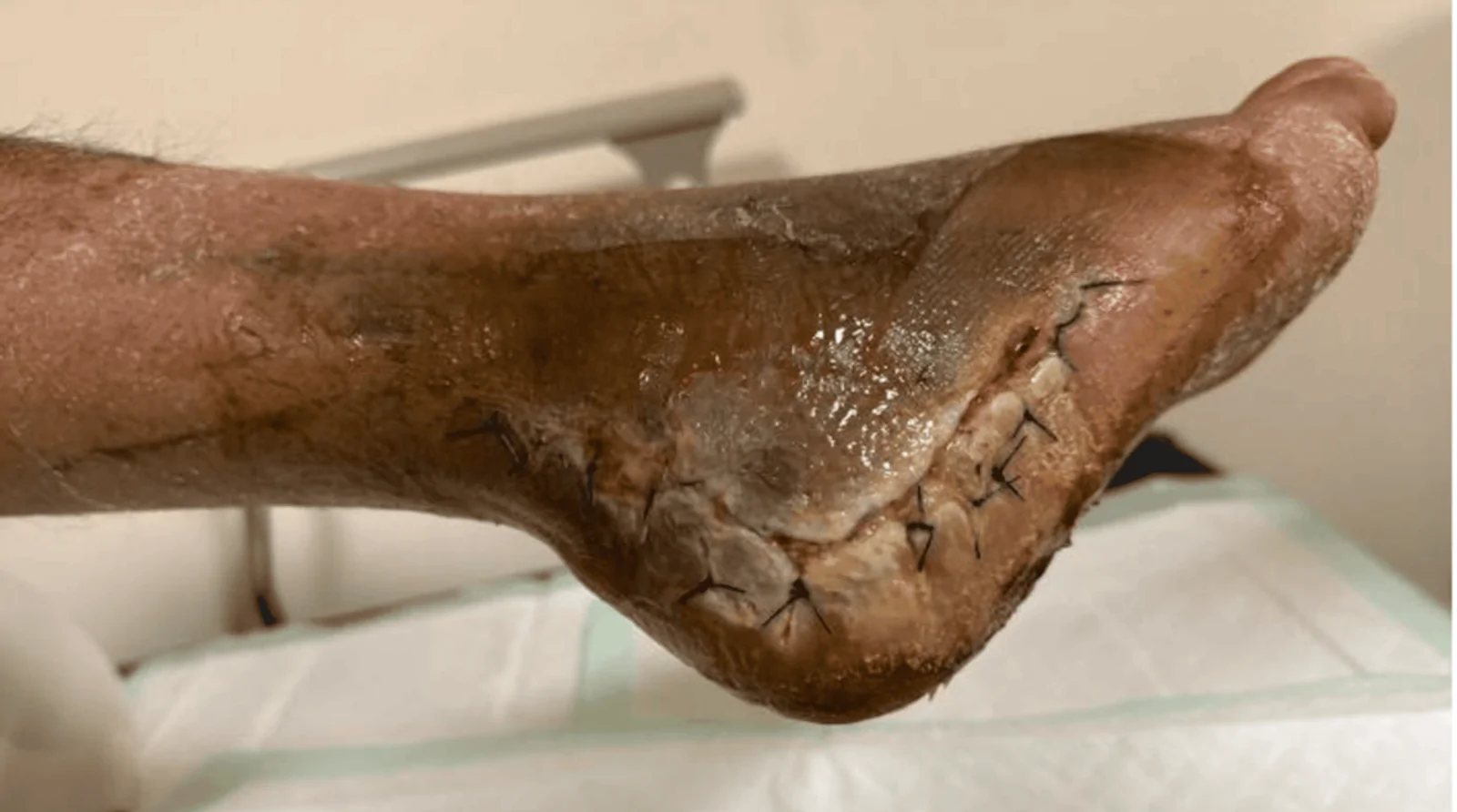
Surgical site infection two weeks after ORIF, demonstrating wound discoloration, purulent discharge, and foul odor. ORIF: open reduction and internal fixation.

The patient was readmitted in May 2025 for emergency irrigation and surgical debridement. All devitalized and infected tissue was excised, and the wound was thoroughly irrigated with 10 L of normal saline containing gentamicin to reduce bacterial burden. Following debridement, a clean wound bed measuring approximately 2 × 2 cm, with dorsal and distal pockets, was obtained (Figure 2).

**Figure 2:**
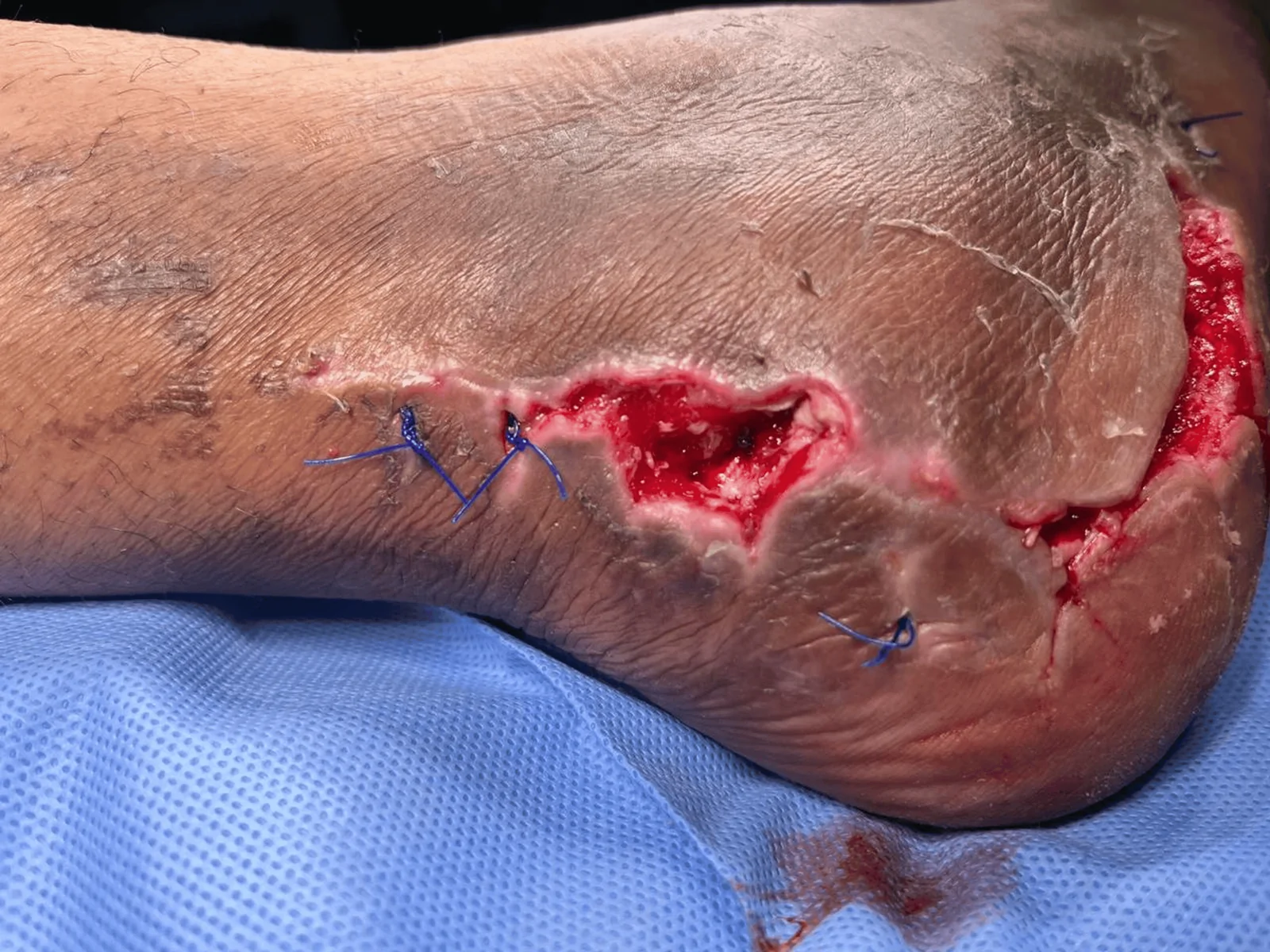
Wound following surgical debridement and irrigation with normal saline and gentamicin, demonstrating complete removal of necrotic tissue and a clean wound bed.

Following complete debridement, 300 mg of MicroMatrix® urinary bladder matrix (UBM) powder was applied to the wound bed (Figure 3). A 7 × 10 cm Cytal® extracellular matrix sheet was then trimmed to fit and placed within the wound to provide a scaffold for tissue regeneration (Figure 4).

**Figure 3:**
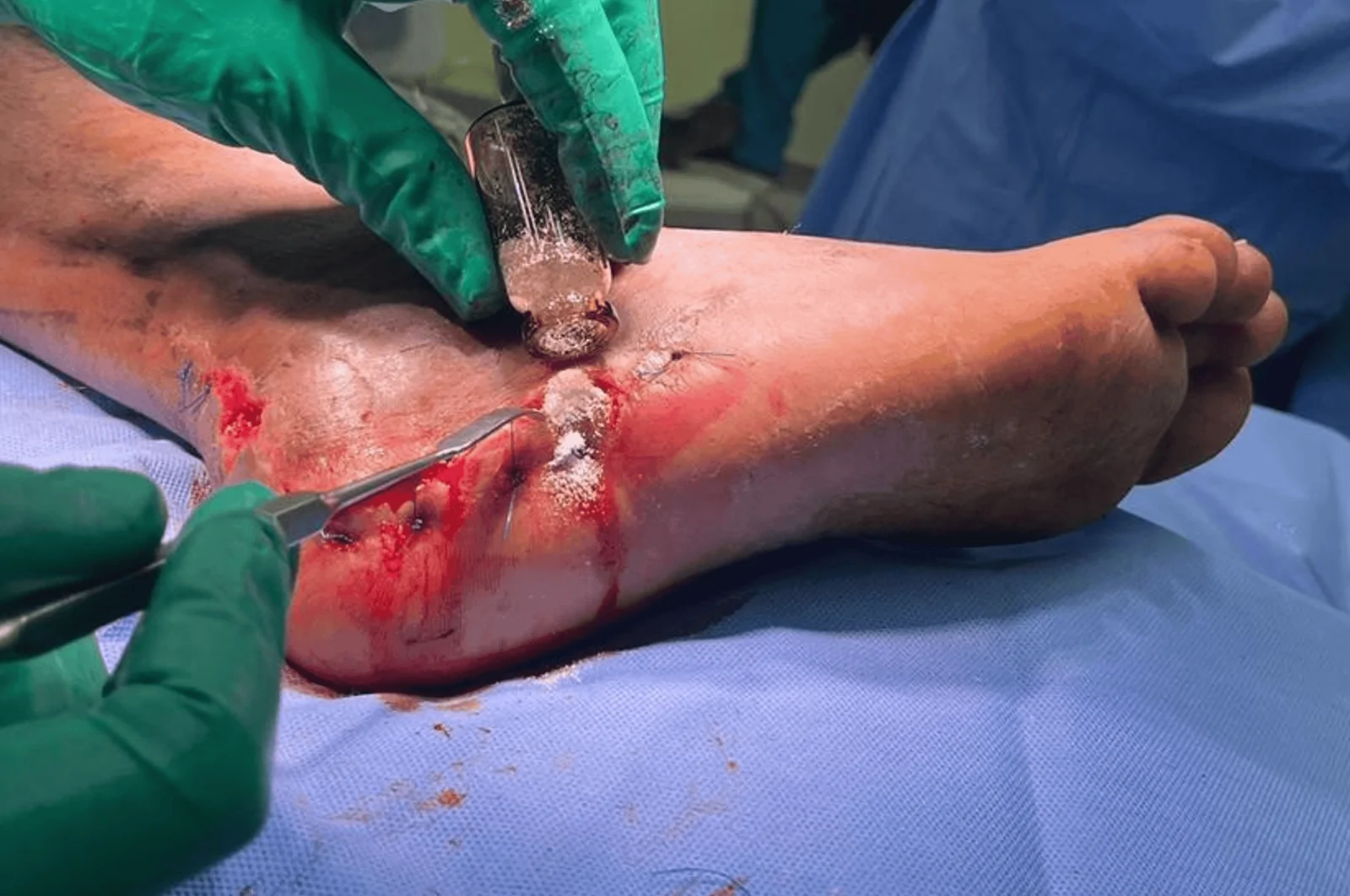
Application of 300 mg of MicroMatrix® (Integra LifeSciences Corp., Princeton, NJ, USA) urinary bladder matrix powder to the wound bed.

**Figure 4:**
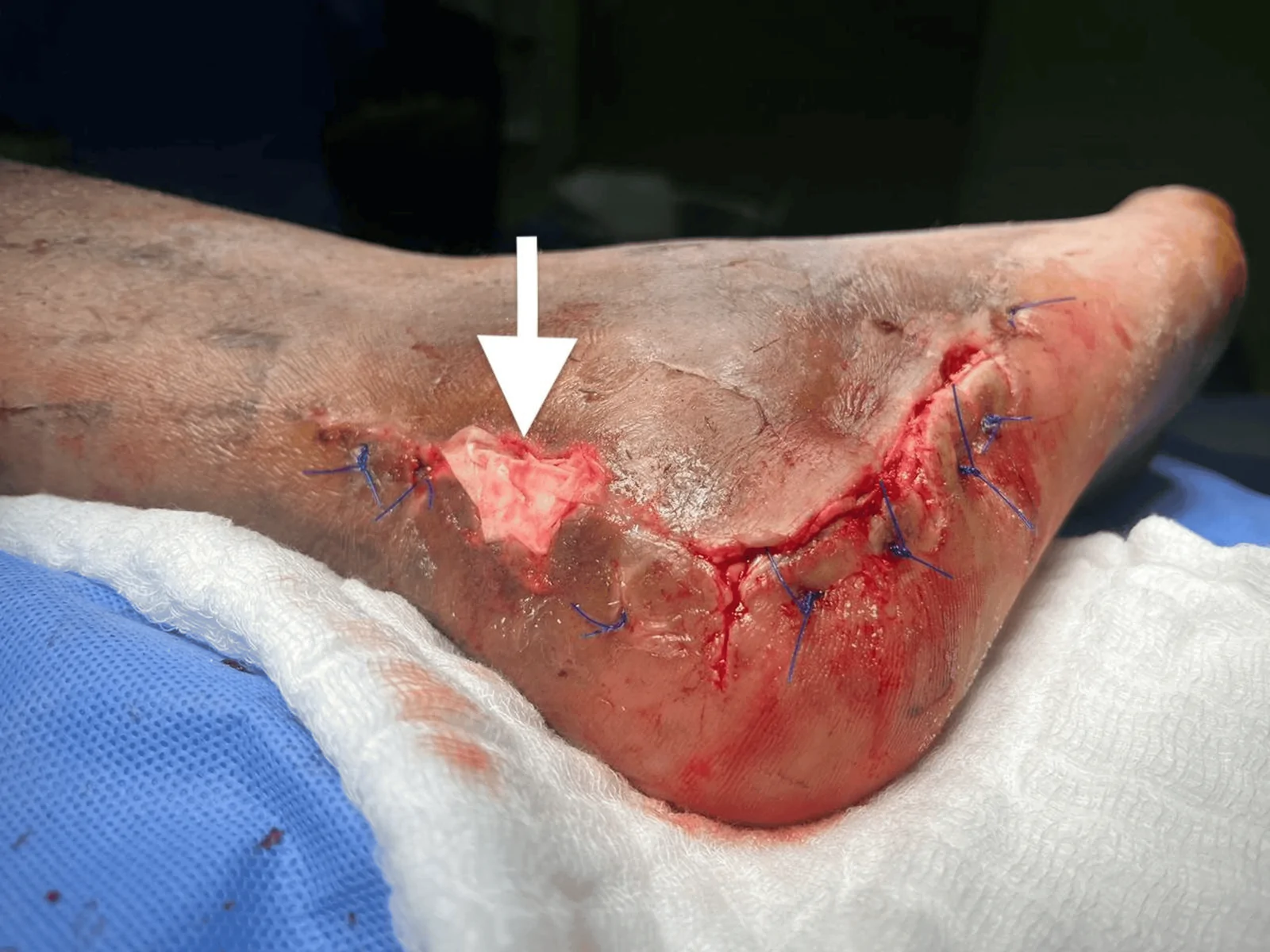
Placement of a trimmed Cytal® (Integra LifeSciences Corp., Princeton, NJ, USA) extracellular matrix sheet within the wound to provide structural support for tissue regeneration.

Deep tissue specimens obtained during debridement were submitted for culture and antimicrobial susceptibility testing. Cultures yielded *Enterobacter cloacae*. The patient remained hospitalized for two days, during which he received intravenous cefepime (2 g every 12 hours) and cefazolin (1 g every 8 hours). He was then discharged on culture-directed antibiotic therapy.

At the first postoperative review, two days after UBM application, marked granulation tissue formation was observed (Figure 5), suggesting early progression of wound healing.

**Figure 5:**
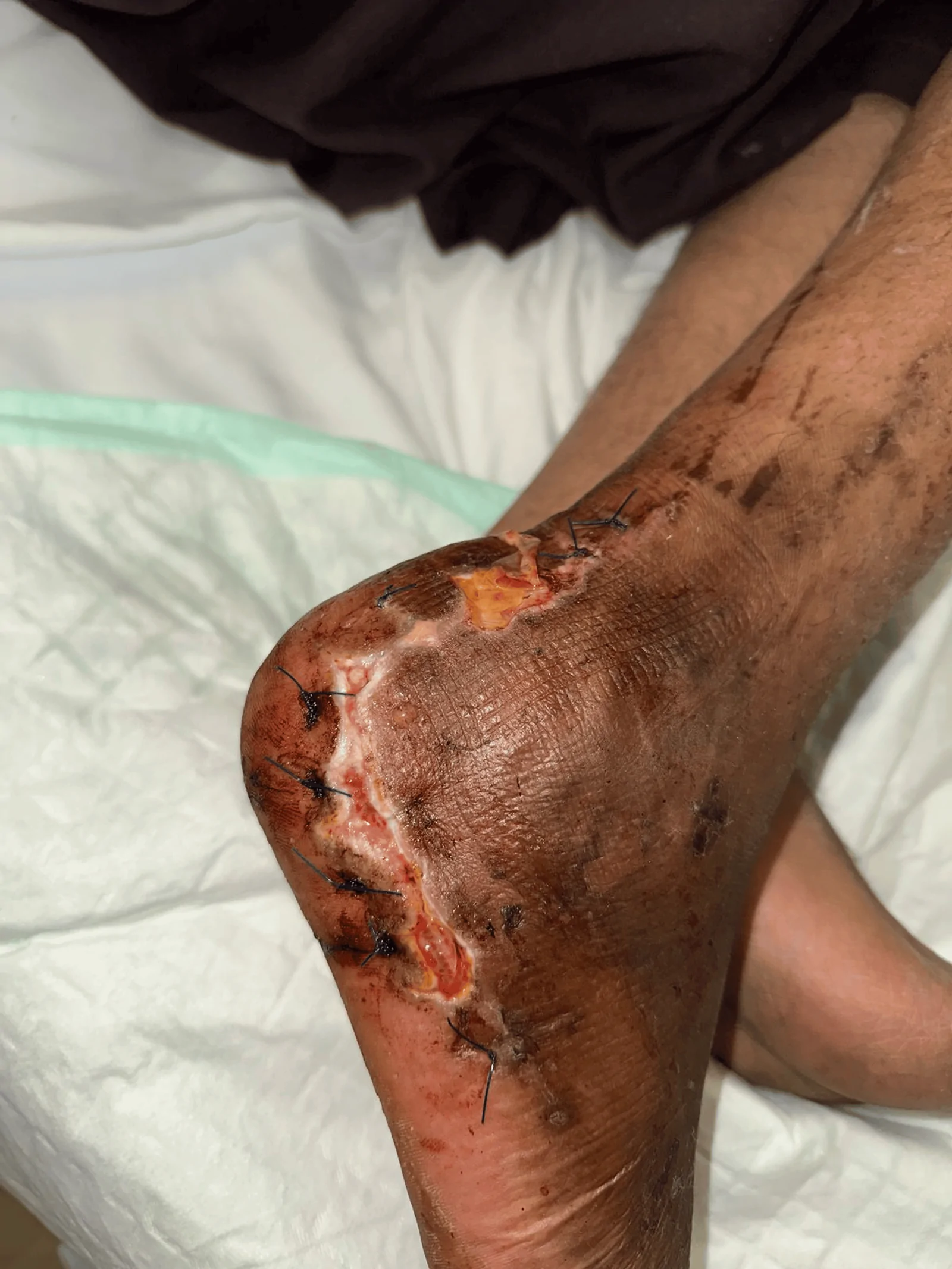
Robust granulation tissue observed two days after the application of MicroMatrix® and Cytal®, indicating rapid tissue proliferation.

The patient was discharged in May 2025, at his own request, with prescriptions for prophylactic enoxaparin (40 mg subcutaneously once daily), pantoprazole (20 mg orally once daily), and trimethoprim-sulfamethoxazole (800/160 mg twice daily). At the subsequent clinic visit in May 2025, wound maceration was observed, which was attributed to poor adherence to postoperative wound care instructions (Figure 6). To support wound healing, hyperbaric oxygen therapy was initiated, and a further 200 mg of MicroMatrix® powder was applied. A silver hydrofiber dressing was used as the primary dressing to manage wound moisture and minimize further maceration.

**Figure 6:**
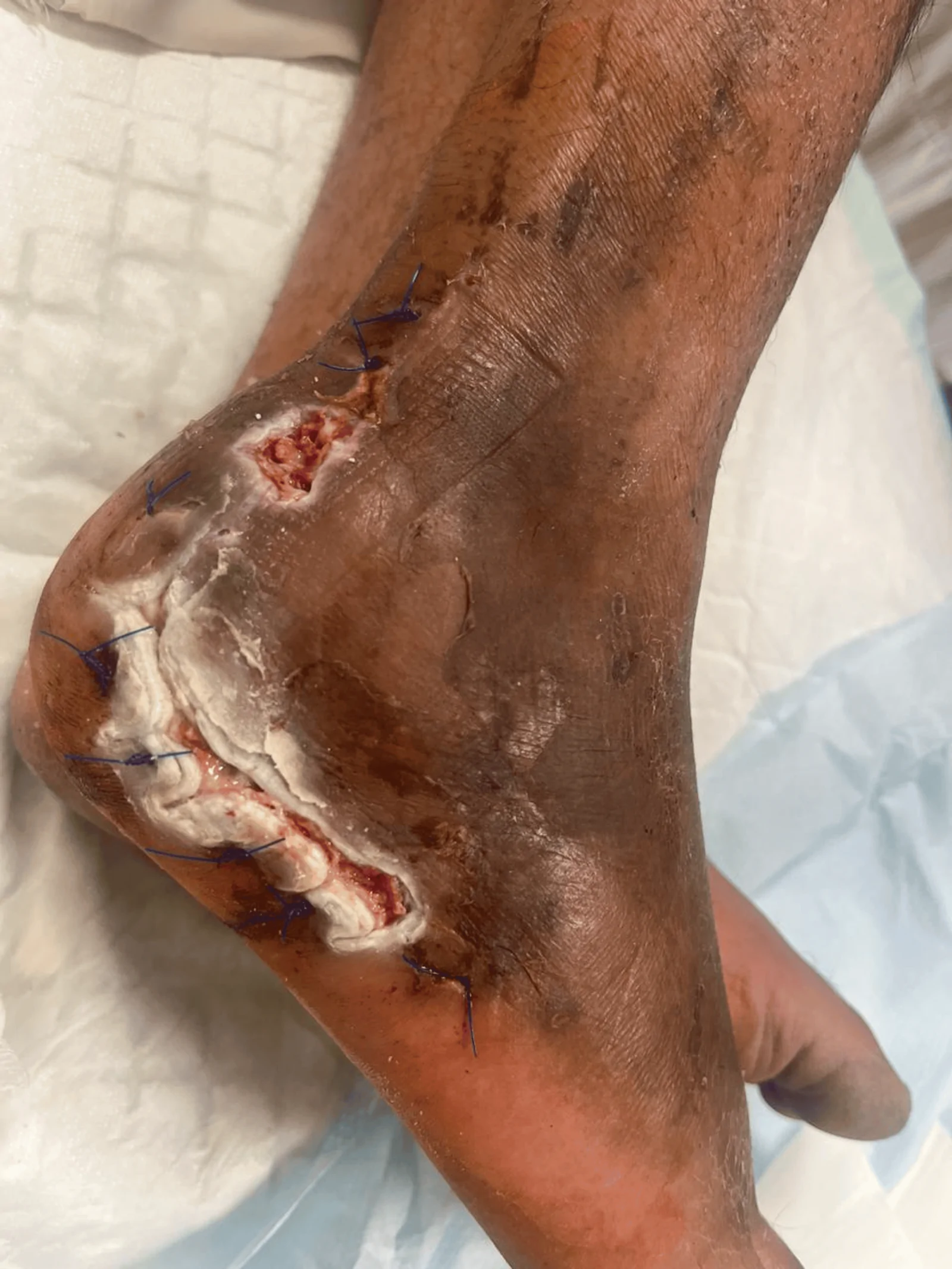
Wound maceration resulting from poor adherence to postoperative wound care. Hyperbaric oxygen therapy and reapplication of MicroMatrix® were subsequently performed.

Telephone follow-up between outpatient visits was implemented to reinforce wound care instructions and improve adherence to treatment. At the next follow-up in May 2025, satisfactory wound healing was observed, and no further application of MicroMatrix® was deemed necessary. Subsequent wound management consisted of regular dressing changes using a non-adherent dressing and gauze, while the posterior backslab was maintained until complete healing was achieved (Figure 7).

**Figure 7:**
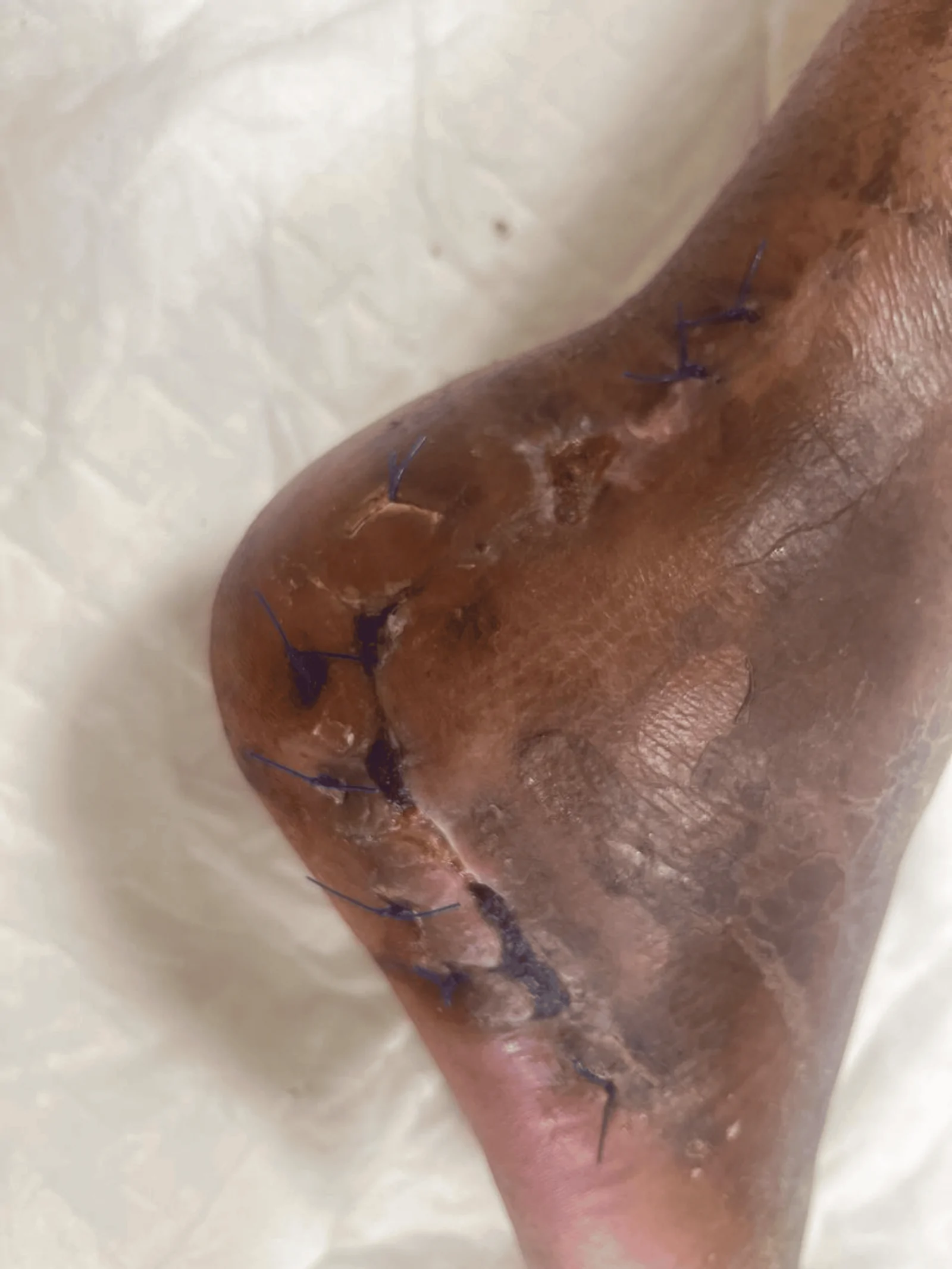
Continued wound healing following discontinuation of MicroMatrix® application, managed with non-adherent dressings and routine wound care.

Progressive wound contraction and epithelialization continued throughout follow-up without further complications. Complete wound healing was achieved within 55 days of initiating UBM therapy, and the sutures were successfully removed (Figure 8). The healed wound showed evidence of satisfactory tissue remodeling, with no recurrent infection, wound dehiscence, or need for further surgical intervention.

**Figure 8:**
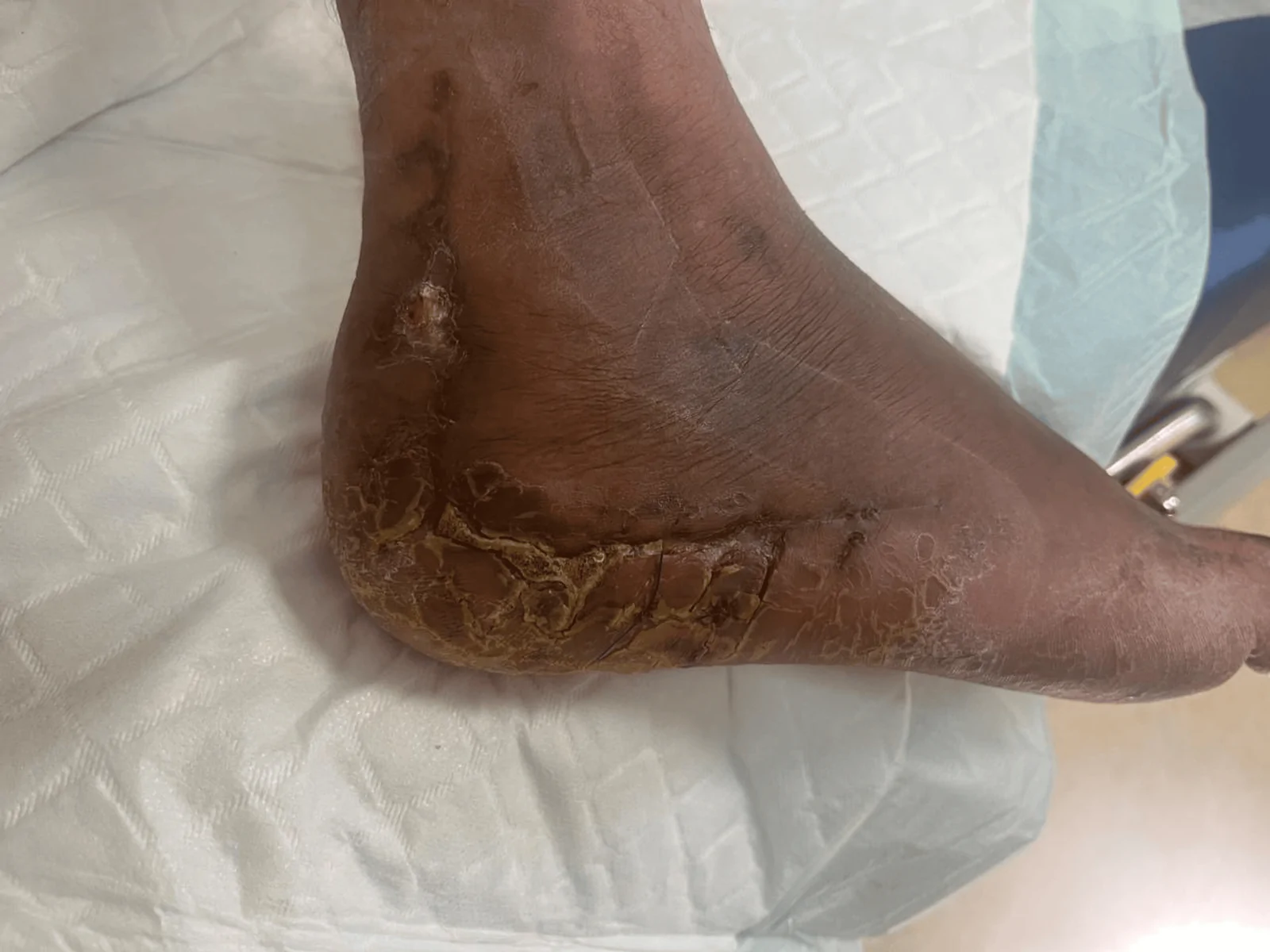
Complete wound healing at the final follow-up, with successful suture removal and no evidence of recurrent infection.

## Discussion

Wound-healing complications following calcaneal fracture surgery remain a significant challenge for both patients and surgeons. These fractures are associated with a substantial risk of postoperative wound complications because of the limited soft tissue coverage of the heel and the extensive soft tissue dissection that may be required during ORIF. In the present case, the patient developed an SSI approximately three weeks after surgery. This complication is consistent with the recognized risk of postoperative wound problems following ORIF for intra-articular calcaneal fractures [4].

A favorable clinical outcome was achieved following thorough surgical debridement and irrigation, followed by the application of urinary bladder matrix (UBM) products, specifically MicroMatrix® and Cytal®. UBM was incorporated into the wound management strategy as an extracellular matrix (ECM)-based scaffold intended to support tissue repair. Within two days of application, substantial granulation tissue formation was observed, suggesting early progression of wound healing. UBM may support tissue repair by providing a scaffold for cellular infiltration and tissue remodeling. Its biological effects have been attributed, in part, to ECM-derived bioactive peptides, including matricryptins, which may modulate inflammation and exhibit antimicrobial activity [7,8].

These observations are consistent with previous reports investigating the biological properties of UBM. Valerio et al. [8] reported that UBM promotes macrophage polarization towards an anti-inflammatory M2 phenotype, potentially supporting tissue repair and limiting persistent inflammation. Similarly, Jiménez-Gastélum et al. [7] described antimicrobial activity associated with collagen-derived peptides, including the inhibition of bacterial growth. These findings provide a biological rationale for investigating UBM in complex wounds, although they do not establish that these mechanisms directly accounted for the clinical outcome in the present case.

Adherence to postoperative wound care instructions was also relevant to the clinical course. At follow-up, wound maceration was observed and attributed to inadequate wound care after discharge. Following reinforcement of wound care instructions, reapplication of MicroMatrix®, and the initiation of adjunctive hyperbaric oxygen therapy, wound healing continued. However, because these interventions were delivered as part of a combined treatment strategy, the individual contribution of UBM to the subsequent improvement cannot be determined from this case alone.

Complete wound closure was achieved within 55 days of initiating UBM therapy, without further surgical intervention. This case illustrates the potential role of UBM as an adjunct to debridement, antimicrobial treatment, and appropriate wound care in managing a complex postoperative wound. Although the outcome is encouraging, a single case cannot establish the safety, efficacy, or cost-effectiveness of UBM relative to alternative treatments. Further studies are needed to clarify its clinical benefits, appropriate indications, and economic value in orthopedic patients at risk of wound complications following calcaneal fracture fixation [8,9].

## Conclusions

This case illustrates the potential role of UBM in managing postoperative wound complications following calcaneal fracture fixation, even in the context of suboptimal adherence to wound care instructions. UBM was used as a scaffold to support tissue regeneration and may promote constructive tissue remodeling through modulation of the local inflammatory response and the release of bioactive peptides with antimicrobial activity. These properties may be beneficial in complex wound management, potentially supporting healing by secondary intention and reducing the need for further surgical debridement.

The findings are consistent with published literature describing the proposed mechanisms of action of UBM, including its potential to promote macrophage polarization towards the pro-regenerative M2 phenotype and its reported antimicrobial activity. These effects may be relevant to two major challenges in complex wound management: infection control and impaired wound healing. In this case, complete wound healing was achieved within 55 days of initiating UBM therapy. However, because UBM was used alongside debridement, antimicrobial treatment, wound care, and hyperbaric oxygen therapy, its individual contribution to healing cannot be established. Its cost-effectiveness also remains to be determined. Further prospective studies and larger clinical trials are warranted to evaluate the effectiveness of UBM in complex wound management and clarify its role across a broader range of orthopedic and surgical wounds.
